# Differential role of CSF fatty acid binding protein 3, α-synuclein, and Alzheimer’s disease core biomarkers in Lewy body disorders and Alzheimer’s dementia

**DOI:** 10.1186/s13195-017-0276-4

**Published:** 2017-07-28

**Authors:** Davide Chiasserini, Leonardo Biscetti, Paolo Eusebi, Nicola Salvadori, Giulia Frattini, Simone Simoni, Naomi De Roeck, Nicola Tambasco, Erik Stoops, Hugo Vanderstichele, Sebastiaan Engelborghs, Brit Mollenhauer, Paolo Calabresi, Lucilla Parnetti

**Affiliations:** 1 Section of Neurology, Department of Medicine, University of Perugia, Perugia, Italy; 2 Oncoproteomics Laboratory, VU University Medical Center, De Boelelaan 1117, 1081HV Amsterdam, The Netherlands; 3 Neurology Clinic, University Hospital S. Maria della Misericordia - University of Perugia, Perugia, Italy; 4 Reference Center for Biological Markers of Dementia (BIODEM), Institute Born-Bunge, University of Antwerp, Antwerp, Belgium; 5 ADx NeuroSciences, Technologiepark 4, 9052 Gent, Belgium; 6 Department of Neurology and Memory Clinic, Hospital Network Antwerp (ZNA) Middelheim and Hoge Beuken, Antwerp, Belgium; 7 Department of Neurosurgery and Institute of Neuropathology, University Medicine Göttingen, Robert-Koch-Str. 40, 37075 Göttingen, Germany; 8 Paracelsus-Elena Klinik, Klinikstrasse 16, 34128 Kassel, Germany; 9 IRRCS S. Lucia Foundation, Rome, Italy

**Keywords:** Cerebrospinal fluid, Biomarkers, Dementia, Heart fatty acid binding protein, Tau, α-Synuclein, Amyloid

## Abstract

**Background:**

Neurodegenerative disorders such as Alzheimer’s disease (AD), Parkinson’s disease with dementia (PDD), and dementia with Lewy bodies (DLB) share clinical and molecular features. Cerebrospinal fluid (CSF) biomarkers may help the characterization of these diseases, improving the differential diagnosis. We evaluated the diagnostic performance of five CSF biomarkers across a well-characterized cohort of patients diagnosed with AD, DLB, PDD, and Parkinson’s disease (PD).

**Methods:**

A total of 208 patients were enrolled in 3 European centers. The diagnostic groups (AD, *n* = 48; DLB, *n* = 40; PDD, *n* = 20; PD, *n* = 54) were compared with cognitively healthy neurological control subjects (patients with other neurological diseases [OND], *n* = 46). CSF levels of fatty acid binding protein 3, heart type (FABP3), α-synuclein (α-syn), amyloid-β peptide 1–42, total tau (t-tau), and phosphorylated tau 181 (p-tau) were assessed with immunoassays. Univariate and multivariate statistical analyses were applied to calculate the diagnostic value of the biomarkers as well as their association with clinical scores.

**Results:**

FABP3 levels were significantly increased in patients with AD and DLB compared with those with PD and OND (*p* < 0.001). CSF t-tau, p-tau, and α-syn were significantly higher in patients with AD than in patients with PDD, DLB, PD, and OND. Combination of FABP3 with p-tau showed high accuracy for the differential diagnosis between AD and DLB (AUC 0.92), whereas patients with AD were separated from those with PDD using a combination of p-tau, FABP3, and α-syn (AUC 0.96). CSF FABP3 was inversely associated with Mini Mental State Examination score in the whole cohort (*r* = −0.42, *p* < 0.001).

**Conclusions:**

The combination of CSF biomarkers linked to different aspects of neurodegeneration, such as FABP3, α-syn, and AD biomarkers, improves the biochemical characterization of AD and Lewy body disorders.

**Electronic supplementary material:**

The online version of this article (doi:10.1186/s13195-017-0276-4) contains supplementary material, which is available to authorized users.

## Background

Neurodegenerative disorders (NDDs) such as Alzheimer’s disease (AD), Parkinson’s disease (PD), and dementia with Lewy bodies (DLB) share a central pathogenic theme: the accumulation, in extra- or intracellular deposits, of aggregated and misfolded proteins [1]. The type of protein, as well as the size, shape, and location of the deposits, is quite typical of each disorder and is used in the pathological examination for characterizing each disease. However, NDDs show remarkable similarities from the clinicopathological point of view, making accurate diagnosis difficult, especially at early stages of the disease [2, 3]. For instance, the clinical presentation of AD and DLB, two diseases considered the most common neurodegenerative forms of dementia, may overlap significantly, leading to low accuracy of the differential diagnosis [4]. Cognitive impairment can occur also in patients initially diagnosed with a prototypical movement disorder such as PD, generally at later stages of disease, and often leading to Parkinson’s disease with dementia (PDD). Apart from the temporal difference in the onset of cognitive deficits, PDD is remarkably similar to DLB in clinical terms, showing, beyond extrapyramidal signs, multidomain impairment and visual hallucinations [5].

Besides clinical similarities, the co-occurrence of different protein aggregates is also a prominent molecular feature of NDDs. On one hand, inclusion bodies composed of α-synuclein (α-syn), representing the major pathological determinants in PD and DLB, can also be detected in AD brains, especially in selected areas such as the amygdala [6]. On the other hand, tau and amyloid-β (Aβ) aggregation, considered the pathological hallmark of AD, is also found in DLB and PD brains, usually to different degrees [7–9].

The presence of similar molecular signatures across NDDs is also detectable in cerebrospinal fluid (CSF) [10]. CSF levels of the Aβ peptide 1–42 (Aβ_1–42_) are generally reduced in both AD and DLB compared with control subjects [11]. Patients with PD may show reduced CSF Aβ_1–42_ levels as well, a decrease often associated with cognitive decline [12, 13]. CSF α-syn is currently studied as a biomarker for PD and other synuclein-associated diseases, generally showing lower levels than control subjects and patients with AD [14–18]. Also, tau proteins in CSF may show a partial overlap between AD and DLB, with phosphorylated tau 181 (p-tau) being the most useful for differential diagnosis [19–21].

Other proteins have been evaluated across NDDs for their potential value in differential diagnosis. Among them, several studies underlined the importance of the fatty acid binding protein 3, heart type (FABP3), a small cytosolic protein involved in lipid transport. In the brain, FABP3 regulates the lipid composition of the membrane [22], entailing possible roles in synapse formation [23] and in the activity of cholinergic and glutamatergic neurons [24]. Increased FABP3 levels were found in the CSF of patients with different neurological disorders, including Creutzfeldt-Jakob disease (CJD), AD in both its prodromal and dementia phases, and vascular dementia (VAD) [25–31]. Furthermore, in CSF, FABP3 strongly correlates with tau, the prototypical marker of neurodegeneration [30]. The role of FABP3 in NDDs related to α-syn aggregation is less defined. Increased levels of FABP3 have been found in the serum of patients diagnosed with DLB and PDD [26]. Furthermore, FABP3 is highly expressed in mouse dopaminergic neurons, and its overexpression has been linked to α-syn aggregation and PD pathogenesis [32].

Considering the different roles of FABP3, total α-syn, and AD core biomarkers across the AD-PD spectrum, we hypothesized that their combination would be of value for the differential diagnosis of NDDs. In this study, we measured this biomarker panel in a large, multicentric cohort composed of patients with AD, DLB, PDD, and PD compared with a group of subjects with other neurological diseases without dementia (OND). Additionally, we explored the associations of the CSF biomarker panel with cognitive decline and other clinical scores in the different diagnostic groups.

## Methods

### Patients and sample collection

A total of 208 subjects were included in this study. One-hundred forty-nine of them were consecutively enrolled at the Center for Memory Disturbances, University Hospital of Perugia (Italy), in the period 2006–2014; 20 patients were from the Reference Center for Biological Markers of Dementia, Institute Born-Bunge, University of Antwerp (Belgium), and 39 were from the Paracelsus-Elena-Klinik, Kassel (Germany). Details on the number of patients per condition are reported in Table 1. The AD group was composed of 48 patients diagnosed with probable AD according to National Institute of Aging-Alzheimer Association criteria [33]. The patients with PD (*n* = 54) were diagnosed with PD according to United Kingdom Brain Bank Society (UKBBS) criteria [34, 35]. Patients with PDD (*n* = 20) were diagnosed according to UKBBS criteria and criteria of the *Diagnostic and Statistical Manual of Mental Disorders, Fourth Edition, Text Revision*. The diagnosis of DLB was made according to McKeith’s criteria [36] in all the centers.

**Table 1:** Demographics and clinical features of the patient cohort

Demographics	AD	DLB	OND	PD	PDD	*p* Value
*N*	48	40	46	54	20	
Sex, male, *n* (%)	23 (47.9)	27 (67.5)	18 (39.1)	35 (64.8)	13 (65.0)	0.026
Age, years	70.9 (9.6)	73.1 (6.3)	58.6 (17.3)	66.0 (8.9)	73.7 (5.7)	<0.001
MMSE score	18.4 (5.2)	19.2 (5.5)	26.7 (4.3)	27.8 (2.2)	19.2 (4.4)	<0.001
MoCA score	–	–	–	25.5 (3.3)	–	–
UPDRS-III score	–	27.9 (12.5)	–	24.3 (12.9)	33.3 (15.9)	0.092
H&Y score	–	3.0 (0.7)	–	1.7 (0.5)	3.6 (1.1)	<0.001
Center	Perugia	20 Kassel, 20 Antwerp	Perugia	Perugia	19 Kassel, 1 Perugia	

*Abbreviations: AD* Alzheimer’s disease, *DLB* Dementia with Lewy bodies, *H&Y* Hoehn & Yahr, *MMSE* Mini Mental State Examination, *MoCA* Montreal Cognitive Assessment, *PD* Parkinson’s disease, *PDD* Parkinson’s disease with dementia, *UPDRS-III* Unified Parkinson’s Disease Rating Scale part III

The number of patients included in each group, age, and sex, together with cognitive and motor scores, are reported. All data are reported using mean (SD), with the exception of the number of male patients, which is reported as a percentage. The *p* value is relative to the nonparametric analysis of variance test for the overall difference among the groups

As neurological controls, 46 subjects who underwent lumbar puncture (LP) for diagnostic reasons but without clinical evidence of dementia were enrolled (OND). The commonest OND diagnoses were headache, epilepsy, psychiatric disorders, and white matter lesions. All the diagnoses of the subjects with OND are reported in Additional file 1. The exclusion criteria for the control group were dementia disorders, atypical parkinsonism (i.e., multiple system atrophy, corticobasal syndrome, progressive supranuclear palsy), and systemic and neoplastic diseases.

The patients underwent a thorough clinical examination by experienced neurologists, including the following: (1) a neuropsychological evaluation, including screening tools such as the Mini Mental State Examination (MMSE) and the Montreal Cognitive Assessment, and extended cognitive batteries for the assessment of memory, language, attention, and executive functions; (2) evaluation of behavioral changes, functional status, and dementia staging by using the Neuropsychiatric Inventory, basic/instrumental activities daily living, and Clinical Dementia Rating; (3) brain magnetic resonance imaging; and (4) blood and CSF analysis. Patients included in the PD, PDD, and DLB groups were also evaluated by means of the Unified Parkinson’s Disease Rating Scale part III (UPDRS-III) and Hoehn and Yahr (H&Y) scores. The LP was performed from 8:00 a.m. to 10:00 a.m. after overnight fasting, following a standardized procedure and according to international guidelines [37]. CSF (10–12 ml) was taken from the L3-L4 or L4-L5 interspace, immediately collected in sterile polypropylene tubes, and gently mixed to avoid possible gradient effects. In Perugia and Kassel, the samples were centrifuged at 2000 × *g* for 10 minutes, aliquoted, and stored at −80 °C. CSF samples from Antwerp were collected in polypropylene vials, immediately frozen in liquid nitrogen, and subsequently stored at −80 °C. Blood-contaminated samples were excluded from the analysis (cutoff of 50 red blood cells per microliter).

### Immunoassays

FABP3, Aβ_1–42_, total tau (t-tau), and p-tau were measured using commercially available enzyme-linked immunosorbent assays (ELISAs) (FABP3 Human ELISA, Hycult Biotech, Uden, The Netherlands; INNOTEST β-AMYLOID(1–42)™, Fujirebio Europe, Gent, Belgium; Total Tau ELISA, Phosphorylated Tau 181 ELISA, EUROIMMUN AG, Lübeck, Germany) and according to previous reports [38, 39]. α-Syn was measured at ADx NeuroSciences (Gent, Belgium) using a new assay developed internally [40]. The ADx α-syn research ELISA is a colorimetry-based sandwich immunoassay (96-well microplate format) with a readout that can be measured in a conventional microplate reader using a 450-nm filter. α-Syn is captured by a C-terminal monoclonal antibody ADx301 (amino acid region 115–125). The (undiluted) sample or calibrator (recombinant full-length α-syn) and the detector monoclonal antibody ADx302 (amino acid region 95–110) are incubated simultaneously for 3 h at room temperature. After a subsequent wash step, addition of streptavidin-peroxidase, and then substrate incubation and reaction stop, the analyte concentration in the samples is calculated using a four-parameter logistic curve fitting the seven nonzero calibrator points (100–5000 pg/ml). Operators blinded to the diagnosis performed the measurements. CSF pools of patients with a positive (AD pool) or negative (non-AD pool) profile for the core AD biomarkers (Aβ_1–42_, t-tau, and p-tau) were run in each plate to check for run-to-run variability.

### Data analysis

Statistical analyses were performed using R software version 3.1 [41]. Continuous variables were described as mean and SD, whereas categorical variables were reported as count and percent. Distribution of biomarkers was checked for normality with the Shapiro-Wilk test. Owing to nonnormality of the distribution of biomarkers, nonparametric analyses were carried out. All correlations were calculated using Spearman’s rho with the Benjamini-Hochberg correction. Nonparametric analysis of variance was used to compare biomarkers levels across all diagnostic groups, accounting for difference in age distribution; in cases of significant differences, pairwise group comparisons were performed using Tukey’s method. The diagnostic performance was assessed by the AUC of the ROC curve. Cutoff values were calculated using sensitivity and specificity that maximized Youden’s index. Ninety-five percent confidence intervals were calculated for the AUC. A backward elimination method was used for model selection by progressively eliminating predictors with the largest individual *p* value, one at a time at each step in the process, until only significant predictors remained. From these models, by using fitted probabilities, we derived ROC curves as well as estimates of the AUC, sensitivity, and specificity. *p* Values less than 0.05 were considered significant for all the analyses.

## Results

### Demographical and clinical features

The demographic and clinical data are reported in Table 1. There was a significant difference in the frequency of male sex among the groups (*p* = 0.019), with a higher percentage in the PD, PDD, and DLB groups than in the AD and OND groups. Also, there was a significant difference among groups in terms of age (*p* < 0.001), which was due to the higher mean age in the AD, DLB, and PDD groups than in the PD and OND groups. As expected, in patients with dementia (AD, DLB, and PDD), the MMSE scores were significantly lower than among subjects with PD and OND (*p* < 0.001). No significant difference was detected among subjects with PD, PDD, and DLB with regard to UPDRS-III score, whereas there was a significant difference among these groups in terms of H&Y stage (*p* < 0.001; PDD > DLB > PD).

### Levels of the CSF biomarkers in the diagnostic groups

CSF levels of the five biomarkers in the diagnostic groups are reported in Table 2. Assay variability for Aβ_1–42_, FABP3, and t-tau was in line with previously published reports [30, 39], whereas for ADx p-tau and α-syn, both intra- and interassay coefficients of variation were below 10% (Additional file 2). Considering that our DLB group was composed of patients enrolled in two different centers (Kassel, *n* = 20; Antwerp, *n* = 20), we tested for the existence of possible center effects. There was no significant center effect for any of the measured biomarkers (Mann-Whitney *U* test; data not shown).

**Table 2:** Cerebrospinal fluid levels of the biomarker panel in the diagnostic groups

Biomarker	OND	PD	PDD	DLB	AD	*p* Value
FABP3, pg/ml	521.6 (354.93)	491.5 (231.3)	738.2 (410.0)	836.3 (450.6)^a,b^	896.1 (514.07)^c,d^	<0.001
α-Syn, pg/ml	1628.0 (705.4)	1846.3 (1216.7)	1381.8 (548.8)	1751.1 (1105.3)	2450.8 (871.24)^c,d,e,f^	<0.001
t-tau, pg/ml	225.9 (114.6)	199.8 (73.9)	292.5 (153.5)	356.7 (176.9)^a,b^	669.8 (304.0)^c,d,e,f^	<0.001
p-tau, pg/ml	45.7 (13.0)	44.6 (9.5)	55.0 (18.8)	60.2 (20.9)^b^	106.1 (37.3)^c,d,e,f^	<0.001
Aβ_1–42_, pg/ml	813.0 (348.7)	792.3 (345.4)	533.8 (168.9)^g,h^	562.1 (249.4)^a,b^	465.0 (159.3)^c,d^	<0.001
Aβ_1–42_-positive, <500 pg/ml	7/34 (17.1%)	13/41 (24.1%)	8/11 (42.1%)	15/25 (37.5%)	32/16 (67.7%)	<0.001

*Abbreviations: Aβ*_*1–42*_ Amyloid-β peptide 1–42, *AD* Alzheimer’s disease, *DLB* Dementia with Lewy bodies, *FABP3* Fatty acid binding protein 3, heart type, *OND* Other neurological diseases, *PD* Parkinson’s disease, *PDD* Parkinson’s disease with dementia, *p-tau*_*181*_ Phosphorylated tau 181, *α-syn* α-Synuclein, *t-tau* Total tau

The cerebrospinal fluid levels of all the tested biomarkers are reported. All data are reported as mean (SD). The *p* values in the table are relative to the general nonparametric analysis of variance test (across all groups), whereas the footnote callouts are relative to the within-group comparisons

^a^DLB vs. OND

^b^DLB vs. PD

^c^AD vs. OND

^d^AD vs. PD

^e^AD vs. DLB

^f^AD vs. PDD

^g^PD vs. PDD

^h^PDD vs. OND

**Fig. 1:**
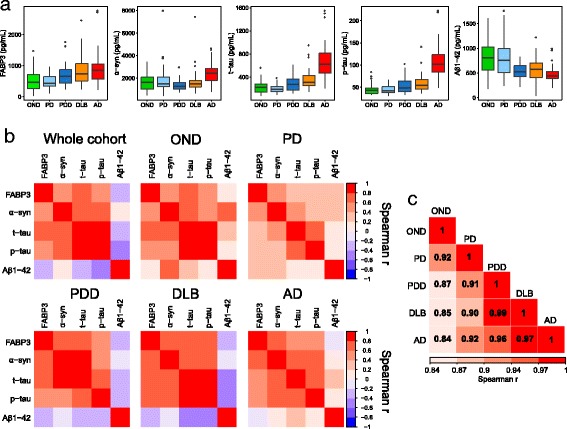
Cerebrospinal fluid (CSF) levels of fatty acid binding protein 3, heart type (FABP3), and tau across the diagnostic groups. **a** Box plots of the five biomarkers across the diagnostic groups. **b**, and **c** Correlation plots (Spearman) for the CSF five-biomarker panel in the whole cohort and in the diagnostic groups. *OND* Other neurological diseases, *PD* Parkinson’s disease, *PDD* Parkinson’s disease with dementia, *DLB* Dementia with Lewy bodies, *AD* Alzheimer’s disease, *Aβ*_*1–42*_ Amyloid-β peptide 1–42, *p-tau* Phosphorylated tau 181, *α-syn* α-Synuclein, *t-tau* Total tau

CSF FABP3 levels (Fig. 1) were significantly higher in the AD group than in the PD and OND groups (*p* < 0.001) (Fig. 1a). Also, patients with DLB showed higher levels of FABP3 than those with PD (*p* < 0.01) and OND (*p* < 0.001). Patients with PDD showed an increase of CSF FABP3 levels compared with subjects with OND and PD, without reaching statistical significance. Subjects with PD and OND showed similar levels of FABP3 in CSF. Aβ_1–42_ was significantly lower in the AD and PDD groups than in the OND and PD groups (Table 2). No significant difference was noted between patients with AD and patients with DLB regarding Aβ_1–42_. Both t-tau and p-tau CSF levels were higher in the AD group than in the DLB, PDD, PD, and OND groups (*p* < 0.001). Furthermore, CSF t-tau levels were significantly higher in the DLB group than in the PD group (*p* < 0.01). α-Syn levels were not significantly different in the PD, PDD, and DLB groups compared with the control group. Interestingly, increased levels of α-syn were found in the AD group compared with the other diagnostic groups and with the OND group (Table 2 and Fig. 1a).

We next explored more deeply the role of Aβ_1–42_ across the diagnostic groups. We dichotomized the cohort according to Aβ_1–42_ positivity with the cutoff used in our clinic for supporting AD diagnosis (500 pg/ml). Interestingly, besides the AD group, there was also an increase in the percentage of the Aβ_1–42_-positive cases in the PD, PDD, and DLB groups compared with the OND group (Table 2). In the whole cohort, FABP3, t-tau, and p-tau were significantly increased in Aβ_1–42_-positive cases (Additional file 3). In the single diagnostic groups, this trend was confirmed but did not reach statistical significance. Instead, there was a significant decrease (approximately 50%) of α-syn CSF levels in subjects with OND and PD who were Aβ_1–42_-positive (Additional file 3).

A complete correlation analysis was carried out for the five biomarkers in the whole cohort and in each diagnostic group (Fig. 1b and Additional file 4). In the whole cohort, FABP3 correlated significantly with t-tau and p-tau (*r* = 0.65, *p* < 0.0001; and *r* =0.58, *p* < 0.001, respectively), confirming previous reports [30]. FABP3 also correlated positively with α-syn (*r* = 0.56, *p* < 0.001), whereas a weak but significant and inverse correlation was found with Aβ_1–42_ (*r* = −0.21, *p* < 0.01). In the whole cohort, we noted a strong correlation between α-syn and tau proteins (*r* = 0.68, *p* < 0.001 for t-tau; *r* = 0.60, *p* < 0.0001 for p-tau), which was generally confirmed also in each diagnostic group to different degrees (Fig. 1b, Additional file 2). When we analyzed the correlations in the single groups, we also calculated the similarity of the biomarker correlation matrices across the different groups. Interestingly, the most similar groups according to the correlation of the CSF biomarkers were PDD and DLB, with a correlation of 0.99, followed by DLB and AD (*r* = 0.97) (Fig. 1c).

**Fig. 2:**
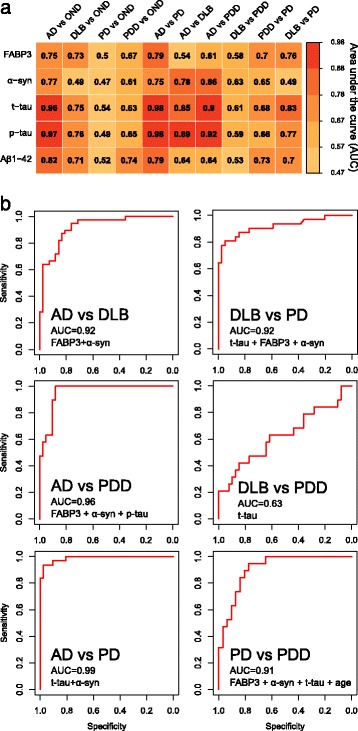
Diagnostic performance of cerebrospinal fluid biomarkers. **a** Heat map of AUCs of the single biomarkers for all diagnostic comparisons. **b** ROC analysis of the logistic regression results for differential diagnosis of neurodegenerative disorders. *Aβ*_*1–42*_ Amyloid-β peptide 1–42, *AD* Alzheimer’s disease, *DLB* Dementia with Lewy bodies, *FABP3* Fatty acid binding protein 3, heart type, *OND* Other neurological diseases, *PD* Parkinson’s disease, *PDD* Parkinson’s disease with dementia, *p-tau* Phosphorylated tau 181, *α-syn* α-Synuclein, *t-tau* Total tau

### Diagnostic performance of the single biomarkers

The diagnostic values of the five biomarkers vs. the control group (OND) and for differential diagnosis were first calculated using univariate ROC analysis. Figure 2a reports the AUC for each of the tested comparisons, and the complete analysis is reported in Additional file 5, including also sensitivity and specificity for each biomarker.

#### Disease vs. control group (OND)

Tau proteins were globally the best biomarkers in distinguishing NDDs from the OND group, reaching a very high accuracy, especially in AD diagnosis (AUC 0.96 for t-tau and 0.97 for p-tau). Indeed, for AD diagnosis vs. subjects with OND, all five biomarkers showed an AUC >0.7, with FABP3 being the biomarker with the lowest performance (AUC 0.75). Four of the five biomarkers (FABP3, t-tau, p-tau, Aβ_1–42_) had a similar accuracy when discriminating patients with DLB from the OND group (AUC approximately 0.7), whereas no biomarker was able to achieve an adequate discrimination of PD from OND (t-tau AUC 0.54). For patients with PDD vs. subjects with OND, the decrease of Aβ_1–42_ was the best biomarker (AUC 0.74).

#### Differential diagnosis among neurodegenerative disorders

Clinically, the differential diagnosis between AD and DLB is of utmost importance. In our cohort, tau proteins confirmed their importance in the characterization of these two dementias. p-tau was the best single biomarker (AUC 0.89), followed by t-tau and α-syn (AUC 0.85 and 0.78, respectively), with all of them increased in AD. A similar pattern was found for the comparison AD vs. PDD (Fig. 2a and Additional file 5). ROC analysis confirmed the similarity between DLB and PDD groups, with no biomarkers showing very high accuracy in distinguishing the two diseases; a decrease of α-syn in the PDD group showed the best performance, reaching an AUC of 0.63 with a specificity of 69% and a sensitivity of 65%. In the comparison between the DLB and PD groups, t-tau was the best biomarker, with an AUC of 0.83, followed by p-tau, FABP3, and Aβ_1–42_, all of them with AUCs ranging from 0.77 to 0.70. Finally, Aβ_1–42_ confirmed its important role in the differentiation between the PDD and PD groups [12], showing an AUC of 0.73 (decreased in PDD), followed by FABP3 (AUC 0.70, increased in PDD).

### Combination of biomarkers for the differential diagnosis of NDDs

To further assess both the effect of potential confounders such as age and the value of biomarker combinations, a multivariate logistic regression approach was used. Several biomarker combinations were tested in different models. Table 3 shows a summary of the biomarkers retained by the best model for each comparison, while in Fig. 2b the ROC analysis of the best model for the differential diagnosis of NDDs is depicted. For the differentiation between AD and OND groups, the best model retained the core CSF AD biomarkers (Aβ_1–42_, t-tau, p-tau) and resulted in the correct classification of 98% of the subjects with a specificity of 88% and a sensitivity of 100%. DLB diagnosis vs. OND was related to CSF Aβ_1–42_ and age; the model was able to correctly classify 79% of the subjects with a specificity of 90% and sensitivity of 69%. The logistic regression analysis of patients with PD vs. subjects with OND included t-tau and age in the final model, correctly classifying 72% of the subjects with a specificity of 58% and a sensitivity of 90%. Also, in the comparison between patients with PDD and the OND group, age played a significant role, together with Aβ_1–42_; this model resulted in the correct classification of 81% of the subjects (specificity 69%, sensitivity 95%).

**Table 3:** Multivariate regression models for the diagnosis of neurodegenerative disorders

Group comparisons	Biomarkers	AUC (95% CI)	Specificity	Sensitivity
AD vs. OND	t-tau, p-tau, Aβ_1–42_	0.98 (0.96–1.00)	0.88	1.00
DLB vs. OND	Age, Aβ_1–42_	0.79 (0.66–0.92)	0.90	0.69
PD vs. OND	Age, t-tau	0.72 (0.57–0.87)	0.58	0.90
PDD vs. OND	Age, Aβ_1–42_	0.81 (0.68–0.94)	0.69	0.95
AD vs. DLB	p-tau, FABP3	0.92 (0.86–0.98)	0.76	0.95
AD vs. PDD	p-tau, a-syn, FABP3	0.96 (0.91–1.00)	0.88	1.00
AD vs. PD	t-tau, α-syn	0.99 (0.97–1.00)	0.97	0.93
DLB vs. PDD	t-tau	0.63 (0.46–0.79)	0.85	0.42
DLB vs. PD	t-tau, a-syn, FABP3	0.92 (0.84–0.99)	0.95	0.80
PD vs. PDD	Age, t-tau, a-syn, FABP3	0.91 (0.84–0.99)	0.77	0.95

*Abbreviations: Aβ*_*1–42*_ Amyloid-β peptide 1–42, *AD* Alzheimer’s disease, *DLB* Dementia with Lewy bodies, *FABP3* Fatty acid binding protein 3, heart type, *OND* Other neurological diseases, *PD* Parkinson’s disease, *PDD* Parkinson’s disease with dementia, *p-tau*_*181*_ Phosphorylated tau 181, *α-syn* α-Synuclein, *t-tau* Total tau

We used logistic regression to analyze which combination of biomarkers would be most useful to distinguish each disease from the control group (OND) and for differential diagnosis. Demographic variables such as age and sex were included to assess their influence on the final models. Several models were fitted using different combination of biomarkers and potential confounders; only the best performing model for each comparison is reported, according to AUC, sensitivity, and specificity

The differential diagnosis of AD from DLB was again dependent on p-tau; however, in the final model, FABP3 was also retained, improving the AUC up to 0.92 but with no significant difference compared with the univariate analysis (*p* = 0.283) (Table 3 and Fig. 2b). A similar improvement in diagnostic accuracy was found for the comparison of patients with AD and patients with PDD, where the inclusion of p-tau, α-syn, and FABP3 led to an AUC of 0.96 with a specificity of 88% and a sensitivity of 100%. Also, the multivariate model was not significantly different from the model including only p-tau (*p* = 0.256 by DeLong test).

Differential diagnosis across Lewy body disorders was significantly improved by our biomarker panel. The inclusion of FABP3 and α-syn for the differentiation of DLB from PD led to an AUC of 0.92 (*p* = 0.022 vs. t-tau alone). A significant improvement was also noted for the comparison between PD and PDD (Table 3) (*p* = 0.017 vs. Aβ_1–42_ alone). The latter was also the only comparison in which age played a significant role and was included in the final model. This was expected because dementia is a feature of PD at later stages, and patients with PDD in our cohort were significantly older than those with PD. The exception for differential diagnosis across Lewy body disorders, was the comparison of DLB vs. PDD, where our panel of biomarkers obtained accuracy similar to that of the univariate analysis (Table 3 and Additional file 5).

### Correlation of the CSF biomarkers with clinical scores

**Fig. 3:**
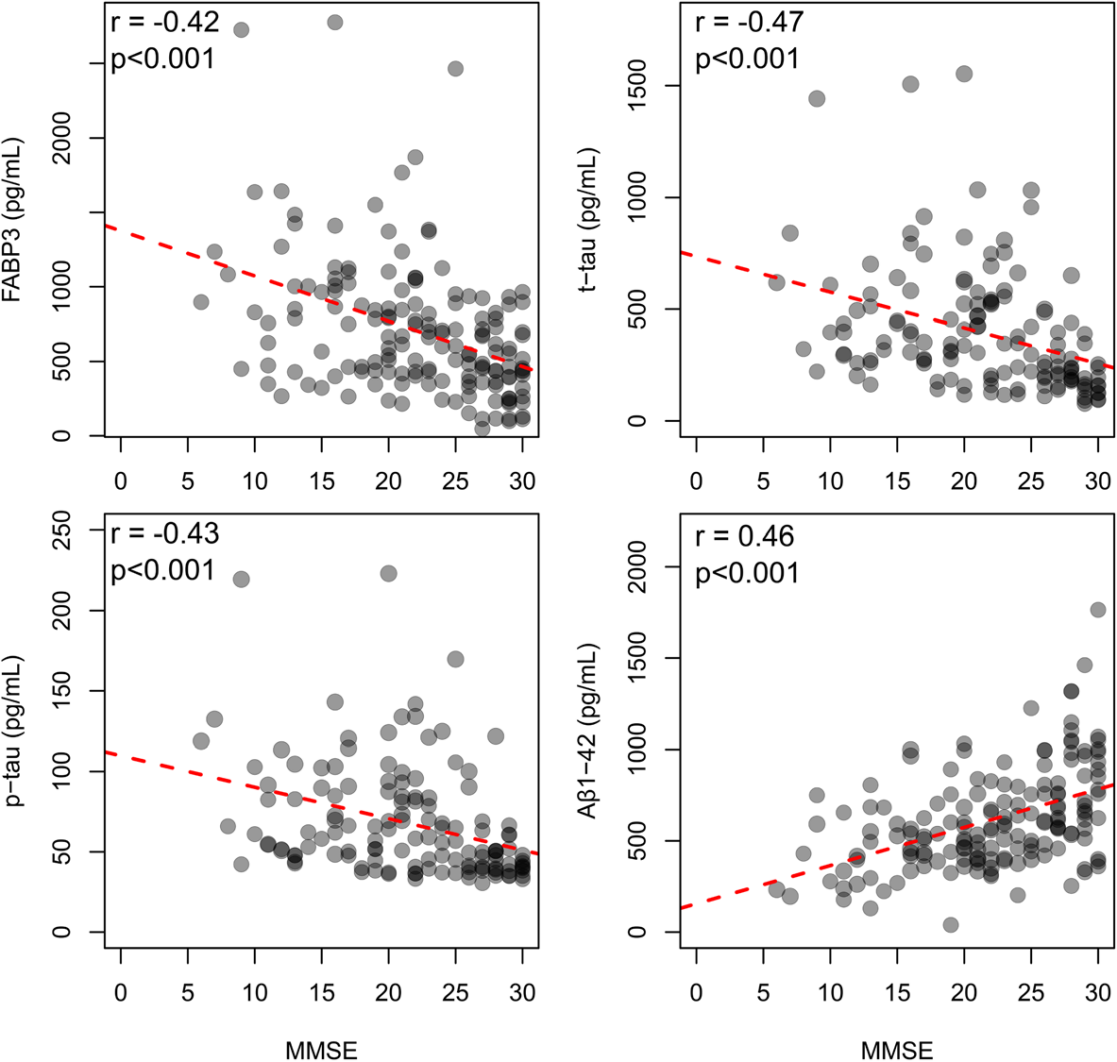
Correlation of cerebrospinal fluid (CSF) biomarkers with cognitive decline. CSF fatty acid binding protein 3, heart type (FABP3), total tau (t-tau), phosphorylated tau 181 (p-tau) and amyloid-β peptide 1–42 (Aβ_1–42_) levels significantly correlated with Mini Mental State Examination (MMSE) scores in the whole cohort. Correlations were calculated using Spearman’s rho

The complete correlation analysis between CSF biomarkers and clinical scores is reported in Additional file 6. All biomarkers were associated to various degrees with baseline MMSE scores, with the exception of α-syn. FABP3 inversely correlated with MMSE score in the whole cohort (*r* = −0.42, *p* < 0.001) (Fig. 3). Tau proteins were strongly and negatively associated with baseline MMSE score (*r* = −0.47 for t-tau, *r* = −0.43 for p-tau, *p* < 0.001), whereas Aβ _1–42_ correlated positively (*r* = 0.46, *p* < 0.001) (Fig. 3). Motor (UPDRS-III and H&Y) scores were available for the PD, PDD, and DLB groups. The correlations of the CSF biomarkers with motor scores were generally weaker than the correlations with cognitive scores. In the whole cohort, FABP3 and t-tau were weakly associated with the H&Y score (*r* = 0.26, *p* < 0.05; and *r* = 0.29, *p* < 0.05, respectively), whereas Aβ_1–42_ was inversely associated with H&Y (*r* = −0.37, *p* < 0.01). In the single diagnostic groups, FABP3 correlated with MMSE only in the PDD group (*r* = −0.49, *p* < 0.05), but after correction for multiple comparisons, no significant correlation was retained, possibly owing to the relatively limited size of the groups.

## Discussion

In this study, we show that the combination of CSF biomarkers linked to different aspects of neurodegeneration may improve the characterization of NDDs, namely AD, DLB, PDD, and PD. In particular, we report the following findings:

1. Tau protein and α-syn levels were significantly increased in patients with AD compared with the other diagnostic groups.
2. FABP3 CSF levels were increased in the AD and DLB groups compared with the OND and PD groups.
3. Combination of FABP3 with p-tau showed excellent performance in the discrimination between AD and DLB, whereas the inclusion of α-syn in the logistic models improved the discrimination of PDD and DLB from PD.
4. FABP3 showed a significant inverse correlation with MMSE score in the whole cohort.

To our knowledge, this is the largest study analyzing the combination of FABP3 and α-syn with AD core biomarkers for differential diagnosis of NDDs, also including patients with PD without dementia. The core CSF AD biomarkers have become an important tool to support the diagnosis of AD and have been included in the new diagnostic criteria for AD [33, 42]. However, these three biomarkers have not shown enough accuracy in the differential diagnosis of dementias [43]. This is possibly related to the existence of a disease continuum across the neurodegeneration spectrum, at both the molecular and phenotypical levels [2].

Our results show that tau proteins are fundamental biomarkers, not only for distinguishing patients with AD from those with OND but also in differential diagnosis across dementias. In our cohort, tau proteins showed a different degree of increase in dementia groups (AD > DLB > PDD). This trend has been found previously, with patients with AD generally showing higher CSF levels of tau proteins than patients with DLB and patients with PDD [43–45]. On one hand, our data show a high discriminative power of p-tau in distinguishing patients with AD from those with DLB and patients with PDD, confirming previous results [46, 47]. On the other hand, researchers in some studies have found substantial overlap of the CSF AD profile between subjects with DLB and AD [11, 44, 48–50]. Because of the current uncertainty about the real potential of classical AD biomarkers in the differential diagnosis of NDDs, the inclusion of additional biomarkers with the core AD panel is mandatory in order to improve neurochemical dementia diagnostics.

α-Syn is currently studied for its possible value as a PD biomarker and in the differential diagnosis of NDDs [51–53]. Researchers in previous studies found that α-syn species, also in combination with tau proteins, may be useful in improving diagnostic accuracy across dementia disorders, especially for DLB [14, 49, 50]. In our cohort, total α-syn levels were not significantly changed between the OND group and the Lewy body disorders groups, showing only a trend toward reduction in patients with PDD and patients with DLB. However, we found significantly increased levels of α-syn in patients with AD, and the final models for differential diagnosis of PD and PDD vs. AD included α-syn. This finding confirms the potential role of α-syn as a biomarker also in AD, where α-syn CSF levels are usually increased compared with those of control subjects and patients with parkinsonism. The alteration of α-syn in AD has been linked to synaptic damage [54] or to an underlying Lewy body pathology [55, 56]. In our cohort, the strong correlation between α-syn and tau proteins without specific group differences may underline an association with neuronal damage, as recently reported [57].

FABP3 measurement in serum and CSF has previously been tested as a biomarker for the differential diagnosis of NDDs, including DLB and PDD, in relatively small-scale studies [26, 58]. In the present study, increased FABP3 CSF levels were linked to AD and DLB, whereas patients with PD showed levels similar to those of subjects with OND. The value of FABP3 as an AD biomarker was moderate compared with the core AD biomarkers, confirming the results of a recent meta-analysis [59]. Previous studies have shown a high correlation between FABP3 and tau proteins in CSF [30, 31], supporting the role of FABP3 as a neurodegeneration biomarker. This is confirmed by the parallel increase in FABP3 and tau in other conditions, such as CJD [25], subarachnoid hemorrhage [60], and VAD [61]. However, some findings may endorse specific roles of FABP3 in AD dementia development, because elevated CSF FABP3 levels have been shown to correlate with atrophy of the entorhinal cortex and amyloid pathology in AD-vulnerable brain regions [62]. The association with amyloid pathology was also found in our study, where CSF levels of FABP3 and tau proteins were increased in Aβ_1–42_-positive subjects, similarly to a recent report [63].

Patients with PD without dementia had levels of FABP3 similar to those of the OND group and were characterized only by reduced t-tau CSF levels. In a recent study, Bäckström and colleagues found that high levels of FABP3 and neurofilament light chain protein, together with low Aβ_1–42_, were significantly associated with the development of dementia after 5–9 years of follow-up in a large cohort of patients with PD [64]. In our study, FABP3 CSF levels were inversely correlated with MMSE scores in the whole cohort. The difference from the above-mentioned study may be due to the shorter follow-up available for patients with PD in our cohort (mean 5.2 months, maximum 1 year). Also, although dementia usually occurs in advanced phases [65], not all patients with PD develop dementia along the disease course. In patients with PDD, CSF FABP3 levels showed a trend toward an increase and were inversely correlated with MMSE score. This evidence supports the idea that FABP3 is linked to the neurodegeneration process and cognitive impairment occurring at later stages [26] and is more evident in patients with PDD. Furthermore, the lack of any association with motor and progression scores in patients with PD and patients with PDD may indirectly support the hypothesis of FABP3 as a degenerative marker not linked to pathogenic mechanisms specific to PD.

Despite the high predictive value of the biomarker combinations for the differentiation between AD and the other dementias, the five biomarkers we tested did not improve the distinction between patients with DLB and patients with PDD, with the best biomarker, α-syn, showing a relatively low discriminatory capability (AUC 0.63). This finding, together with the high correlation between the CSF profile of the five biomarkers between DLB and PDD (*r* = 0.99), confirms the molecular and clinical similarities between these two conditions, which can be considered as a continuum across the pathogenesis of Lewy body disorders [66].

Our study has some limitations. Some of the diagnostic groups were enrolled only in one center (AD, OND, and PD in Perugia), possibly introducing a source of variability in the results linked to CSF processing. However, the three clinics are experienced reference centers for CSF biomarker measurement and follow international guidelines for CSF collection [37]. Another limitation is the heterogeneity of the control group, which was composed of different neurological disorders and not of healthy control subjects. Nonetheless, the OND group represents a population ordinarily assayed for CSF biomarkers in a neurology and memory clinic, thus exemplifying the use of CSF biomarkers in routine clinical practice. A third limitation might be related to the disease stage, because most of the patients included in this study were enrolled at quite advanced stages of the disease. Therefore, the value of this panel of biomarkers at early stages of neurodegeneration remains to be determined, even though FABP3 has already shown some diagnostic value in early AD [30, 31].

## Conclusions

Our results show that the inclusion of FABP3 and α-syn in the core panel of AD CSF biomarkers can improve the molecular characterization of NDDs encompassing AD dementia and Lewy body disorders. Researchers in other studies have investigated the combination of different biomarkers for the differential diagnosis of dementia and parkinsonism, highlighting how measuring panels of proteins linked to different facets of neurodegeneration will be essential for the biochemical characterization of NDDs and to support clinical diagnosis [19, 67, 68]. Further longitudinal studies including different cohorts of patients with NDDs are necessary to verify the role of this biomarker panel across the neurodegeneration spectrum.

## Additional files

Additional file 1: Diagnosis of patients with OND. The diagnosis, sex, and age (when available) of each patient with OND are reported. In the OND group, we enrolled, as control subjects, patients diagnosed with other neurological conditions without cognitive impairment who had undergone lumbar puncture for diagnostic reasons. The exclusion criteria for the OND group are reported in the main text. *OND* Other neurological diseases, *NA* Not available. (DOCX 17 kb)

Additional file 2: Variability of ELISAs used in this study. The intra- and interassay coefficients of variation (CVs) are reported. The intra-assay CV was calculated using duplicate values of two internal controls, whereas the inter-assay CVs derive from five different runs in different plates of the same internal controls. (DOCX 14 kb)

Additional file 3: Influence of amyloid positivity on CSF biomarker levels. The patients were divided into two groups according to Aβ_1–42_ CSF levels. A cutoff of 500 pg/ml was used for Aβ_1–42_, corresponding to the internal cutoff used in our clinic. The levels of the CSF biomarkers were compared in each diagnostic group and in the whole cohort. An increase of FABP3, t-tau, and p-tau was noted in the whole cohort. *FABP3* Fatty acid binding protein 3, heart type, *t-tau* Total tau, *p-tau* Phosphorylated tau 181, *α-syn* α-Synuclein. (DOCX 18 kb)

Additional file 4: Correlation matrix of the CSF biomarker panel. Correlations among CSF biomarkers were calculated according to Spearman’s correlation. Spearman’s rho and corresponding *p* values are reported. (DOCX 16 kb)

Additional file 5: ROC analysis of the CSF biomarkers for the different comparisons. The diagnostic performance of each biomarker was calculated according to ROC analysis. AUC, sensitivity, and specificity, together with the 95% CI of each parameter, are included. *AD* Alzheimer’s disease, *PD* Parkinson’s disease, *PDD* Parkinson’s disease with dementia, *DLB* Dementia with Lewy bodies, *OND* Other neurological diseases. (DOCX 16 kb)

Additional file 6: Spearman’s correlations adjusted with Benjamini-Hochberg correction between biomarkers and clinical parameters in the whole cohort and within each group. (DOCX 21 kb)

## Abbreviations

AD: Alzheimer’s disease
Aβ_1–42_: Amyloid-β peptide 1–42
CJD: Creutzfeldt-Jakob disease
CSF: Cerebrospinal fluid
DLB: Dementia with Lewy bodies
ELISA: Enzyme-linked immunosorbent assay
FABP3: Fatty acid binding protein 3, heart type
H&Y: Hoehn and Yahr scale
LP: Lumbar puncture
MMSE: Mini Mental State Examination
MoCA: Montreal Cognitive Assessment
NDD: Neurodegenerative disorder
OND: Other neurological diseases
PD: Parkinson’s disease
PDD: Parkinson’s disease with dementia
p-tau: Phosphorylated tau 181
t-tau: Total tau
UKBBS: United Kingdom Brain Bank Society
UPDRS-III: Unified Parkinson’s Disease Rating Scale part III
VAD: Vascular dementia
α-syn: α-Synuclein
