# Supplementary material for: Differential role of CSF fatty acid binding protein 3, α-synuclein, and Alzheimer’s disease core biomarkers in Lewy body disorders and Alzheimer’s dementia
Source: Alzheimers Res Ther. 2017 Jul 28;9:52. doi: 10.1186/s13195-017-0276-4 (PMC5532764; doi:10.1186/s13195-017-0276-4)
Supplement: Supplementary file 1 — Diagnosis of patients with OND. The diagnosis, sex, and age (when available) of each patient with OND are reported. In the OND group, we enrolled, as control subjects, patients diagnosed with other neurological conditions without cognitive impairment who had undergone lumbar puncture for diagnostic reasons. The exclusion criteria for the OND group are reported in the main text. OND Other neurological diseases, NA Not available. (DOCX 17 kb) [file 13195_2017_276_MOESM1_ESM.docx]

**Additional file 1. Diagnosis of OND patients**

| **Group** | **Center** | **Sex** | **Age** | **Diagnosis** |
| --- | --- | --- | --- | --- |
| OND | Perugia | M | 65 | Ataxia |
| OND | Perugia | F | 57 | Vertigo / Dizziness |
| OND | Perugia | F | NA | Dystonia |
| OND | Perugia | M | 78 | Dystonia |
| OND | Perugia | M | 64 | Epylepsy |
| OND | Perugia | F | 76 | Epylepsy |
| OND | Perugia | M | 67 | Epylepsy |
| OND | Perugia | M | 57 | Epylepsy |
| OND | Perugia | F | 51 | Epylepsy |
| OND | Perugia | F | 79 | Epylepsy |
| OND | Perugia | M | 68 | Epylepsy |
| OND | Perugia | F | NA | Headache |
| OND | Perugia | F | 48 | Headache |
| OND | Perugia | M | 79 | Headache |
| OND | Perugia | F | 30 | Intracranial Hypertension |
| OND | Perugia | M | 75 | Meningoencephalitis |
| OND | Perugia | F | NA | Metabolic Encephalopathy |
| OND | Perugia | F | 51 | Metabolic Encephalopathy |
| OND | Perugia | M | 72 | Metabolic Encephalopathy |
| OND | Perugia | F | 57 | Miller Fisher Syndrome |
| OND | Perugia | F | NA | Multiple sclerosis |
| OND | Perugia | M | NA | Multiple sclerosis |
| OND | Perugia | M | NA | Multiple sclerosis |
| OND | Perugia | F | 32 | Multiple sclerosis |
| OND | Perugia | F | NA | Psychiatric disorder |
| OND | Perugia | F | 22 | Psychiatric disorder |
| OND | Perugia | F | 22 | Psychiatric disorder |
| OND | Perugia | F | 69 | Psychiatric disorder |
| OND | Perugia | F | NA | Third Cranial Nerve Palsy |
| OND | Perugia | F | NA | Third Cranial Nerve Palsy |
| OND | Perugia | M | NA | Transient ischemic attack/stroke |
| OND | Perugia | F | NA | Transient ischemic attack/stroke |
| OND | Perugia | M | 27 | Transient ischemic attack/stroke |
| OND | Perugia | F | 57 | Transient global amnesia |
| OND | Perugia | F | 63 | Transient global amnesia |
| OND | Perugia | F | 60 | Transient global amnesia |
| OND | Perugia | M | 69 | White Matter Lesions |
| OND | Perugia | M | NA | White Matter Lesions |
| OND | Perugia | M | 62 | White Matter Lesions |
| OND | Perugia | F | 76 | White Matter Lesions |
| OND | Perugia | F | NA | White Matter Lesions |
| OND | Perugia | M | 51 | White Matter Lesions |
| OND | Perugia | M | 52 | White Matter Lesions |
| OND | Perugia | M | 27 | White Matter Lesions |
| OND | Perugia | F | 64 | White Matter Lesions |
| OND | Perugia | M | 78 | White Matter Lesions |

The diagnosis, sex and age (when available) of each OND patient are reported in supplementary table 1. In the OND group, we enrolled as controls non-demented subjects with some different neurological diseases who underwent lumbar puncture for diagnostic reasons. The exclusion criteria for the OND group are reported in the manuscript. OND= other neurological diseases; NA = not available; M = male; F = female
