# Supplementary material for: Differential role of CSF fatty acid binding protein 3, α-synuclein, and Alzheimer’s disease core biomarkers in Lewy body disorders and Alzheimer’s dementia
Source: Alzheimers Res Ther. 2017 Jul 28;9:52. doi: 10.1186/s13195-017-0276-4 (PMC5532764; doi:10.1186/s13195-017-0276-4)
Supplement: Supplementary file 2 — Variability of ELISAs used in this study. The intra- and interassay coefficients of variation (CVs) are reported. The intra-assay CV was calculated using duplicate values of two internal controls, whereas the inter-assay CVs derive from five different runs in different plates of the same internal controls. (DOCX 14 kb) [file 13195_2017_276_MOESM2_ESM.docx]

**Additional file 2. Variability of ELISA assays used in this study.**

| **Biomarker** | **Intra-assay CV (%)** | **Inter-assay CV (%)*** |
| --- | --- | --- |
| FABP3 | 3.6 | 11.8 |
| Aβ1-42 | 3.2 | 7.2 |
| α-syn | 2.6 | 1.7 |
| t-tau | 7.7 | 5.1 |
| p-tau | 4.7 | 4.6 |

The intra- and inter-assay coefficients of variation (CV) are reported. The intra-assay CV was calculated on duplicate values of two internal controls, while the inter-assay CVs derive from 5 different runs in different plates of the same internal controls.
