# Supplementary material for: Differential role of CSF fatty acid binding protein 3, α-synuclein, and Alzheimer’s disease core biomarkers in Lewy body disorders and Alzheimer’s dementia
Source: Alzheimers Res Ther. 2017 Jul 28;9:52. doi: 10.1186/s13195-017-0276-4 (PMC5532764; doi:10.1186/s13195-017-0276-4)
Supplement: Supplementary file 3 — Influence of amyloid positivity on CSF biomarker levels. The patients were divided into two groups according to Aβ1–42 CSF levels. A cutoff of 500 pg/ml was used for Aβ1–42, corresponding to the internal cutoff used in our clinic. The levels of the CSF biomarkers were compared in each diagnostic group and in the whole cohort. An increase of FABP3, t-tau, and p-tau was noted in the whole cohort. FABP3 Fatty acid binding protein 3, heart type, t-tau Total tau, p-tau Phosphorylated tau 181, α-syn α-Synuclein. (DOCX 18 kb) [file 13195_2017_276_MOESM3_ESM.docx]

**Additional file 3. Influence of amyloid positivity on CSF biomarker levels.**

| Group | **Biomarker** | **n** | **Negative** | **n** | **Positive** | **p-value** |
| --- | --- | --- | --- | --- | --- | --- |
| Whole cohort | FABP3 (pg/mL) | 124 | 636.74 (404.57) | 74 | 788.18 (462.88) | 0.014 |
|  | α-syn (pg/mL) | 109 | 1923.48 (1100.74) | 68 | 1845.09 (905.88) | 0.866 |
|  | t-tau (pg/mL) | 102 | 326.74 (257.20) | 66 | 437.47 (261.11) | 0.001 |
|  | p-tau (pg/mL) | 99 | 57.44 (28.73) | 65 | 78.33 (37.10) | 0.001 |
| OND | FABP3 (pg/mL) | 31 | 530.18 (339.90) | 7 | 628.32 (451.79) | 0.685 |
|  | α-syn (pg/mL) | 24 | 1845.77 (708.83) | 7 | 1063.79 (501.08) | 0.011 |
|  | t-tau (pg/mL) | 22 | 235.14 (119.25) | 7 | 225.98 (127.81) | 0.941 |
|  | p-tau (pg/mL) | 20 | 45.31 (11.12) | 7 | 51.79 (18.85) | 0.85 |
| PD | FABP3 (pg/mL) | 41 | 507.45 (228.26) | 12 | 436.88 (243.64) | 0.225 |
|  | α-syn (pg/mL) | 35 | 2006.18 (1307.40) | 9 | 1224.51 (382.30) | 0.02 |
|  | t-tau (pg/mL) | 29 | 197.39 (67.46) | 9 | 207.53 (96.23) | 0.813 |
|  | p-tau (pg/mL) | 28 | 43.70 (9.16) | 8 | 47.93 (10.54) | 0.358 |
| PDD | FABP3 (pg/mL) | 11 | 608.40 (240.49) | 8 | 905.38 (560.51) | 0.442 |
|  | α-syn (pg/mL) | 11 | 1272.90 (419.85) | 8 | 1501.64 (722.84) | 0.6 |
|  | t-tau (pg/mL) | 11 | 248.86 (134.29) | 8 | 339.28 (176.47) | 0.238 |
|  | p-tau (pg/mL) | 11 | 49.26 (12.75) | 8 | 62.21 (24.77) | 0.31 |
| DLB | FABP3 (pg/mL) | 25 | 810.81 (491.39) | 15 | 878.74 (385.55) | 0.346 |
|  | α-syn (pg/mL) | 24 | 1821.48 (1348.16) | 15 | 1638.61 (554.86) | 0.721 |
|  | t-tau (pg/mL) | 25 | 340.63 (193.83) | 15 | 383.55 (146.67) | 0.125 |
|  | p-tau (pg/mL) | 25 | 57.01 (17.66) | 15 | 65.43 (25.25) | 0.28 |
| AD | FABP3 (pg/mL) | 16 | 922.05 (583.27) | 32 | 883.14 (485.32) | 0.957 |
|  | α-syn (pg/mL) | 15 | 2495.10 (728.35) | 29 | 2427.82 (948.08) | 0.624 |
|  | t-tau (pg/mL) | 15 | 745.13 (359.17) | 27 | 627.99 (266.78) | 0.405 |
|  | p-tau (pg/mL) | 15 | 106.01 (40.35) | 27 | 106.16 (36.24) | 0.897 |

The patients were divided according in two groups according to Aβ1-42 CSF levels. A cut-off of 500 pg/mL was used for Aβ1-42, corresponding to the internal cut-off used in our clinic. The levels of the CSF biomarkers were compared in each diagnostic group and in the whole cohort. An increase of FABP3 , t-tau and p-tau was noticed in the whole cohort. FABP3 = fatty acid binding protein 3, heart, t-tau= total tau, p-tau = phosphorylated tau 181, α-syn = α-synuclein, n = number of patients.
