# Supplementary material for: Differential role of CSF fatty acid binding protein 3, α-synuclein, and Alzheimer’s disease core biomarkers in Lewy body disorders and Alzheimer’s dementia
Source: Alzheimers Res Ther. 2017 Jul 28;9:52. doi: 10.1186/s13195-017-0276-4 (PMC5532764; doi:10.1186/s13195-017-0276-4)
Supplement: Supplementary file 4 — Correlation matrix of the CSF biomarker panel. Correlations among CSF biomarkers were calculated according to Spearman’s correlation. Spearman’s rho and corresponding p values are reported. (DOCX 16 kb) [file 13195_2017_276_MOESM4_ESM.docx]

**Additional file 4. Correlation matrix of the CSF biomarker panel**

| Group | Biomarkers | Spearman Rho | | | | p-value | | | |
| --- | --- | --- | --- | --- | --- | --- | --- | --- | --- |
|  |  | FABP3 | α-syn | t-tau | p-tau | FABP3 | α-syn | t-tau | p-tau |
| Whole cohort | FABP3 | 1.00 |  |  |  | NA |  |  |  |
|  | α-syn | 0.56 | 1.00 |  |  | 2.22E-16 | NA |  |  |
|  | t-tau | 0.65 | 0.68 | 1.00 |  | p<0.0001 | p<0.0001 | NA |  |
|  | p-tau | 0.59 | 0.60 | 0.91 | 1.00 | p<0.0001 | p<0.0001 | p<0.0001 | NA |
|  | Aβ1-42 | -0.21 | 0.08 | -0.33 | -0.40 | 3.04E-03 | 2.82E-01 | 9.61E-06 | 9.37E-08 |
| OND | FABP3 | 1.00 |  |  |  | NA |  |  |  |
|  | α-syn | 0.50 | 1.00 |  |  | 2.38E-03 | NA |  |  |
|  | t-tau | 0.65 | 0.62 | 1.00 |  | 4.05E-05 | 1.34E-04 | NA |  |
|  | p-tau | 0.57 | 0.59 | 0.82 | 1.00 | 7.53E-04 | 4.61E-04 | 1.70E-08 | NA |
|  | Aβ1-42 | 0.04 | 0.61 | 0.20 | 0.13 | 8.17E-01 | 2.47E-04 | 2.88E-01 | 5.31E-01 |
| AD | FABP3 | 1.00 |  |  |  | NA |  |  |  |
|  | α-syn | 0.72 | 1.00 |  |  | 4.62E-08 | NA |  |  |
|  | t-tau | 0.59 | 0.70 | 1.00 |  | 4.45E-05 | 3.06E-07 | NA |  |
|  | p-tau | 0.58 | 0.74 | 0.75 | 1.00 | 6.75E-05 | 2.48E-08 | 9.05E-09 | NA |
|  | Aβ1-42 | 0.00 | 0.15 | 0.21 | 0.00 | 9.94E-01 | 3.42E-01 | 1.80E-01 | 9.94E-01 |
| DLB | FABP3 | 1.00 |  |  |  | NA |  |  |  |
|  | α-syn | 0.65 | 1.00 |  |  | 7.33E-06 | NA |  |  |
|  | t-tau | 0.63 | 0.77 | 1.00 |  | 1.10E-05 | 1.17E-08 | NA |  |
|  | p-tau | 0.62 | 0.60 | 0.92 | 1.00 | 2.34E-05 | 4.54E-05 | p<0.0001 | NA |
|  | Aβ1-42 | -0.27 | -0.11 | -0.41 | -0.40 | 9.58E-02 | 4.94E-01 | 7.93E-03 | 1.03E-02 |
| PD | FABP3 | 1.00 |  |  |  | NA |  |  |  |
|  | α-syn | 0.51 | 1.00 |  |  | 4.52E-04 | NA |  |  |
|  | t-tau | 0.39 | 0.50 | 1.00 |  | 1.67E-02 | 1.54E-03 | NA |  |
|  | p-tau | 0.28 | 0.32 | 0.70 | 1.00 | 1.01E-01 | 5.77E-02 | 4.31E-06 | NA |
|  | Aβ1-42 | 0.21 | 0.32 | 0.07 | 0.06 | 1.27E-01 | 3.50E-02 | 6.97E-01 | 7.38E-01 |
| PDD | FABP3 | 1.00 |  |  |  | NA |  |  |  |
|  | α-syn | 0.78 | 1.00 |  |  | 4.15E-05 | NA |  |  |
|  | t-tau | 0.69 | 0.81 | 1.00 |  | 6.75E-04 | 1.78E-05 | NA |  |
|  | p-tau | 0.47 | 0.54 | 0.78 | 1.00 | 3.55E-02 | 1.37E-02 | 4.86E-05 | NA |
|  | Aβ1-42 | -0.24 | -0.08 | -0.38 | -0.31 | 3.18E-01 | 7.53E-01 | 1.10E-01 | 1.94E-01 |

Correlations among CSF biomarkers were calculated according to Spearman with Benjamini-Hochberg correction. Spearman Rho and corresponding p-values are reported.
