# Supplementary material for: Differential role of CSF fatty acid binding protein 3, α-synuclein, and Alzheimer’s disease core biomarkers in Lewy body disorders and Alzheimer’s dementia
Source: Alzheimers Res Ther. 2017 Jul 28;9:52. doi: 10.1186/s13195-017-0276-4 (PMC5532764; doi:10.1186/s13195-017-0276-4)
Supplement: Supplementary file 5 — ROC analysis of the CSF biomarkers for the different comparisons. The diagnostic performance of each biomarker was calculated according to ROC analysis. AUC, sensitivity, and specificity, together with the 95% CI of each parameter, are included. AD Alzheimer’s disease, PD Parkinson’s disease, PDD Parkinson’s disease with dementia, DLB Dementia with Lewy bodies, OND Other neurological diseases. (DOCX 16 kb) [file 13195_2017_276_MOESM5_ESM.docx]

**Additional file 5. ROC analysis of the CSF biomarkers for the different comparisons**

| **Comparison** | **Biomarker** | **AUC** | **95% CI AUC** | **Specificity** | **Sensitivity** | **Cut-off** |
| --- | --- | --- | --- | --- | --- | --- |
| AD vs. DLB | FABP3 | 0.5404 | 0.4173-0.6634 | 0.50 | 0.68 | 852.2 |
|  | α-syn | 0.7841 | 0.6797-0.8885 | 0.84 | 0.69 | 1672.3 |
|  | t-tau | 0.8524 | 0.7675-0.9372 | 0.79 | 0.83 | 461.4 |
|  | **p-tau** | **0.8914** | **0.8185-0.9642** | **0.90** | **0.78** | **69.2** |
|  | Aβ1-42 | 0.6432 | 0.5183-0.7682 | 0.79 | 0.60 | 537.0 |
| AD vs. OND | FABP3 | 0.7534 | 0.6528-0.854 | 0.58 | 0.86 | 783.6 |
|  | α-syn | 0.7734 | 0.6691-0.8777 | 0.68 | 0.80 | 2132.1 |
|  | t-tau | 0.9603 | 0.9209-0.9997 | 0.93 | 0.88 | 346.7 |
|  | **p-tau** | **0.9731** | **0.9446-1.0000** | **0.98** | **0.84** | **50.6** |
|  | Aβ1-42 | 0.8227 | 0.7273-0.918 | 0.92 | 0.68 | 612.0 |
| AD vs. PD | FABP3 | 0.7862 | 0.6971-0.8752 | 0.73 | 0.72 | 609.5 |
|  | α-syn | 0.7474 | 0.641-0.8538 | 0.68 | 0.80 | 2126.4 |
|  | **t-tau** | **0.9843** | **0.9645-1.0000** | **0.88** | **1.00** | **394.1** |
|  | p-tau | 0.9792 | 0.9555-1.0000 | 0.90 | 1.00 | 68.0 |
|  | Aβ1-42 | 0.789 | 0.6961-0.8818 | 0.92 | 0.70 | 611.5 |
| AD vs. PDD | FABP3 | 0.6135 | 0.4631-0.7639 | 0.54 | 0.75 | 833.9 |
|  | α-syn | 0.8625 | 0.7647-0.9603 | 0.84 | 0.80 | 1665.0 |
|  | t-tau | 0.9024 | 0.822-0.9827 | 0.86 | 0.85 | 416.5 |
|  | **p-tau** | **0.9202** | **0.8475-0.993** | **0.90** | **0.90** | **69.3** |
|  | Aβ1-42 | 0.6431 | 0.4807-0.8055 | 0.65 | 0.68 | 486.5 |
| DLB vs. OND | FABP3 | 0.7267 | 0.6186-0.8349 | 0.70 | 0.65 | 616.1 |
|  | α-syn | 0.4905 | 0.3518-0.6291 | 0.79 | 0.37 | 1911.1 |
|  | t-tau | 0.7538 | 0.6414-0.8662 | 0.63 | 0.82 | 288.0 |
|  | **p-tau** | **0.7593** | **0.6434-0.8751** | **0.65** | **0.84** | **50.7** |
|  | Aβ1-42 | 0.7137 | 0.6011-0.8263 | 0.85 | 0.56 | 757.5 |
| DLB vs. PD | FABP3 | 0.7608 | 0.6635-0.8582 | 0.70 | 0.74 | 621.2 |
|  | α-syn | 0.4883 | 0.3623-0.6144 | 0.92 | 0.20 | 1042.6 |
|  | **t-tau** | **0.827** | **0.7342-0.9197** | **0.75** | **0.84** | **256.4** |
|  | p-tau | 0.7677 | 0.6619-0.8735 | 0.80 | 0.64 | 44.5 |
|  | Aβ1-42 | 0.7005 | 0.5946-0.8063 | 0.78 | 0.59 | 715.0 |
| PDD vs. DLB | FABP3 | 0.575 | 0.4167-0.7333 | 0.70 | 0.50 | 612.9 |
|  | **α-syn** | **0.6321** | **0.4744-0.7897** | **0.69** | **0.65** | **1323.7** |
|  | t-tau | 0.61 | 0.4458-0.7742 | 0.85 | 0.40 | 210.2 |
|  | p-tau | 0.5913 | 0.4288-0.7537 | 0.88 | 0.35 | 41.5 |
|  | Aβ1-42 | 0.5342 | 0.3825-0.686 | 0.33 | 0.84 | 676.0 |
| PD vs. OND | FABP3 | 0.4969 | 0.375-0.6189 | 0.44 | 0.66 | 529.0 |
|  | α-syn | 0.4692 | 0.3378-0.6005 | 0.49 | 0.59 | 1644.4 |
|  | **t-tau** | **0.5435** | **0.4012-0.6857** | **0.36** | **0.87** | **263.1** |
|  | p-tau | 0.4866 | 0.3427-0.6304 | 0.55 | 0.56 | 42.0 |
|  | Aβ1-42 | 0.5185 | 0.3995-0.6376 | 0.54 | 0.56 | 789.0 |
| PDD vs. OND | FABP3 | 0.6651 | 0.5251-0.8052 | 0.40 | 0.90 | 375.7 |
|  | α-syn | 0.6079 | 0.455-0.7607 | 0.49 | 0.80 | 1652.4 |
|  | t-tau | 0.6295 | 0.4716-0.7875 | 0.85 | 0.40 | 321.7 |
|  | p-tau | 0.6516 | 0.4931-0.8102 | 0.87 | 0.45 | 57.4 |
|  | **Aβ1-42** | **0.7439** | **0.6213-0.8665** | **0.59** | **0.89** | **715.0** |
| PD vs. PDD | FABP3 | 0.6962 | 0.5581-0.8344 | 0.43 | 0.90 | 402.0 |
|  | α-syn | 0.6455 | 0.5-0.7909 | 0.68 | 0.65 | 1334.7 |
|  | t-tau | 0.6763 | 0.5131-0.8395 | 0.87 | 0.55 | 267.4 |
|  | p-tau | 0.6625 | 0.499-0.826 | 0.69 | 0.65 | 47.4 |
|  | **Aβ1-42** | **0.7349** | **0.6205-0.8493** | **0.59** | **0.89** | **710.0** |

The diagnostic performance of each biomarker was calculated according to receiver operator characteristic (ROC) analysis. Area under the curve (AUC), sensitivity and specificity together with the 95% confidence interval and cut-off value of each parameter are included. AD= Alzheimer’s disease; PD= Parkinson’s disease; PDD= Parkinson’s disease with dementia; DLB= Dementia with Lewy Bodies; OND= other neurological diseases
