# Supplementary material for: Differential role of CSF fatty acid binding protein 3, α-synuclein, and Alzheimer’s disease core biomarkers in Lewy body disorders and Alzheimer’s dementia
Source: Alzheimers Res Ther. 2017 Jul 28;9:52. doi: 10.1186/s13195-017-0276-4 (PMC5532764; doi:10.1186/s13195-017-0276-4)
Supplement: Supplementary file 6 — Spearman’s correlations adjusted with Benjamini-Hochberg correction between biomarkers and clinical parameters in the whole cohort and within each group. (DOCX 21 kb) [file 13195_2017_276_MOESM6_ESM.docx]

**Additional file 6.** Spearman’s correlations adjusted with Benjamini-Hochberg correction between biomarkers and clinical parameters in the whole cohort and within each group.

| **Group comparison** | **Biomarker** | **Age^1^** | **MMSE^2^** | **UPDRS-III^5^** | **H&Y^5^** |
| --- | --- | --- | --- | --- | --- |
| Whole cohort | FABP3 | 0.38*** | -0.42*** | 0.09 | 0.26* |
|  | α-syn | 0.11 | -0.06 | -0.1 | -0.18 |
|  | t-tau | 0.33*** | -0.47*** | 0.07 | 0.29* |
|  | p-tau | 0.31*** | -0.43*** | -0.06 | 0.16 |
|  | Aβ1-42 | -0.15* | 0.46*** | -0.25 | -0.37** |
| OND | FABP3 | 0.56*** | -0.37 | - | - |
|  | α-syn | 0.09 | -0.04 | - | - |
|  | t-tau | 0.34 | -0.55 | - | - |
|  | p-tau | 0.35 | -0.29 | - | - |
|  | Aβ1-42 | -0.07 | 0.00 | - | - |
| PD | FABP3 | 0.27 | 0.05 | 0.18 | 0.16 |
|  | α-syn | 0.07 | 0.04 | 0.21 | 0.17 |
|  | t-tau | 0.43 | -0.28 | 0.43 | 0.47 |
|  | p-tau | 0.36 | 0.1 | 0.11 | 0.11 |
|  | Aβ1-42 | -0.05 | 0.21 | -0.21 | -0.25 |
| PDD | FABP3 | 0.21 | -0.49 | -0.3 | 0.29 |
|  | α-syn | 0.32 | -0.34 | -0.34 | 0.01 |
|  | t-tau | 0.31 | -0.45 | -0.52 | -0.21 |
|  | p-tau | 0.29 | -0.24 | -0.57 | -0.28 |
|  | Aβ1-42 | -0.29 | 0.42 | -0.08 | -0.01 |
| DLB | FABP3 | 0.16 | -0.01 | -0.11 | -0.01 |
|  | α-syn | 0.14 | 0.31 | -0.37 | -0.01 |
|  | t-tau | -0.04 | 0.04 | -0.05 | -0.12 |
|  | p-tau | -0.03 | -0.02 | -0.01 | -0.29 |
|  | Aβ1-42 | 0.19 | 0.31 | -0.25 | 0.24 |
| AD | FABP3 | 0.06 | -0.18 | - | - |
|  | α-syn | 0.04 | 0 | - | - |
|  | t-tau | 0.01 | 0.1 | - | - |
|  | p-tau | -0.04 | 0.04 | - | - |
|  | Aβ1-42 | 0.21 | 0.16 | - | - |

The Spearman’s correlations between cerebrospinal fluid biomarkers and clinical parameters (age, cognitive and motor scores) adjusted with Benjamini-Hochberg correction are reported in supplementary table 3. MMSE= Mini mental state examination; MOCA= Montreal cognitive assessment; UPDRS III= Unified Parkinson’s disease rating score III (motor part); H&Y= Hoehn and Yahr scale. ^1,2^ Data from all patients, ^3^Data from PD and AD - ^4^ Data from PD - ^5^ Data from PD, PDD, DLB.
